# Serum Amyloid A as a Promising Biomarker in Domestic Animals’ Reproduction: Current Knowledge and Future Perspective

**DOI:** 10.3390/ani12050589

**Published:** 2022-02-25

**Authors:** Marilena Bazzano, Andrea Marchegiani, Alessandro Troisi, Amy McLean, Fulvio Laus

**Affiliations:** 1 School of Biosciences and Veterinary Medicine, University of Camerino, 62032 Camerino, Italy; andrea.marchegiani@unicam.it (A.M.); alessandro.troisi@unicam.it (A.T.); fulvio.laus@unicam.it (F.L.); 2 Department of Animal Science, University of California, Davis (UC Davis), Davis, CA 95616, USA; acmclean@ucdavis.edu

**Keywords:** acute phase protein, serum amyloid A, bitch, cow, mare, endometritis, mastitis

## Abstract

**Simple Summary:**

Acute phase proteins (APPs) are useful markers which can be evaluated in animals to assess health status and characterize inflammation, infection, and trauma. Among APPs, serum amyloid A (SAA) has been widely investigated in pets and food-producing species as a possible biomarker of inflammatory and infective conditions, especially in the field of animal reproduction. The aims of this paper are to review the literature available on the use of SAA for the diagnosis and monitoring of inflammatory reproductive disease in animals, critically appraising the usefulness of such marker and summarizing the current state of knowledge.

**Abstract:**

The investigation of acute phase proteins in veterinary medicine has opened the doors towards the identification and use of new markers for a timely assessment of health status in both companion and food-producing animals. The aim of this paper is to review the literature available on the use of serum amyloid A (SAA), an acute phase protein, for the diagnosis and monitoring of reproductive disorders in animals. This review critically appraises the usefulness of such marker in clinical practice and summarizes the current state of knowledge. Recent advances in the diagnosis and monitoring of reproductive diseases are presented, highlighting where SAA evaluation may enhance early diagnostic tools for dogs, cats, cattle, and equines.

## 1. Introduction

An ongoing challenge in both human and veterinary medicine is discovering new biomarkers, for early identification of subclinical disease, while being able to identify healthy and ill animals [1]. Biomarkers are biological molecules (usually proteins) found in cells, tissues, and body fluids (blood, urine, feces, exhaled breath) that can be quantified as indicators of physiological and pathological conditions [2]. Ideally, a biomarker should be easily detectable and effective in both identifying the onset and monitoring the progress/outcome of the disease [2].

Acute phase response (APR) is a systemic and dynamic process, including a wide range of pathophysiological responses, such as fever, leukocytosis, hormone alterations, and muscle protein depletion, which combine to minimize tissue damage while enhancing the repair process [3]. During APR, specific plasma proteins, known as acute-phase proteins (APPs), which are mainly secreted by the liver to provide an early and non-specific protection against insults [3]. In APR, cytokines act as messengers between the site of disorder/disease and the hepatocytes that synthetize the APPs [4].

APPs represent a large and wide-ranging group of serum non-specific proteins, unrelated to immunoglobulins [5]. APPs may increase due to multiple causes unrelated to inflammation, such as a response to transportation, stress, malnourishment, and parturition, as well as other a stress-related causes. Both an increase in APPs and serum glucocorticoid levels occur with such conditions [6]. According to the definition of APPs, the concentration will increase by >25% in response to proinflammatory cytokines. It has been suggested that APPs are biomarkers of inflammation, infection, and trauma in human and veterinary medicine [3,6,7,8]. According to variations in serum when measuring APPs, they can be labelled as “positively reacting” or “negatively reacting”. Positive APPs, such as serum amyloid A (SAA), haptoglobin (Hp), C-reactive protein (CRP), and fibrinogen, show a “major”, “moderate”, and “minor” increase in their serum level upon initiation of APR. Major APPs, such as SAA, Hp, and CRP, are characterized by a concentration usually below 1 μg/mL in healthy animals which dramatically increases up to 1000 fold following an inflammatory stimulus, reaching a peak around 24–48 h and then decreases rapidly upon recovery. Moderate APPs as alpha1 acid glycoprotein (AGP) increase from 5- to 10-fold following an inflammatory stimulus, reaching a peak after 2 or 3 days and then decrease more slowly than other major APPs. Minor APPs, whose utility is still debated in veterinary medicine, gradually increases by 50 to 100% of its quiescent level [7]. Negative APPs, such as albumin and transferrin, decrease during the inflammatory response and, apart from albumin, have limited use in clinical pathology [7].

Among APPs, SAA has been widely investigated in different species, including dogs, cats, horses, and ruminants, as a possible biomarker of inflammatory and infective processes, especially in the field of animal reproduction [9,10] (Table 1 and Table 2).

Reproductive failure is one of the most significant factors that limit the productivity of animal production systems and negatively influence the welfare of pets and their owners [11]. For dogs and cats, subclinical endometritis, endometrial hyperplasia, and pyometra are the most frequently disorders faced in clinical practice [12]. While endometritis is a major cause of subfertility and infertility in horses and cows [13,14,15], subclinical mastitis represents the major reproductive disorder in sheep flock [16]. Since SAA has been evaluated in different aspects of domestic animals’ reproduction, the aim of this paper is to provide an updated review on the clinical relevance of using SAA in the detection and monitoring of inflammatory and infective diseases of reproductive importance in such species.

**Table 1:** Studies about serum amyloid A evaluation in pets, ruminants, and mares.

Species	Animals/Disease/Condition	Reference
DOG	Healthy bitches and bitches with pyometra underwent ovariohysterectomy	Dabrowski et al., 2006 [17]
	Bitches with pyometra, with normal and complicated post-ovariohysterectomy period	Dabrowski et al., 2009 [18]
	Healthy bitches and bitches with pyometra	Hagman et al., 2009 [19]
	Healthy bitches and bitches suffering from systemic inflammation, including pyometra	Christensen et al., 2014 [20]
	Septic and non-septic bitches with pyometra	Jitpean et al., 2014 [21]
	Healthy bitches and bitches with open- and closed cervix pyometra	Dabrowski et al., 2017 [22]
	Mastitis	Kaszak et al., 2018 [23]
	Mammary tumor	Tecles et al., 2009 [24]
CAT	Healthy cats and cats with pyometra	Yuki et al., 2020 [25]
RUMINANTS	Healthy cows and cows with postpartum endometritis	Chan et al., 2009 [26]
	Healthy cows and cows with endometritis	Biswal et al., 2014 [27]
	Healthy cows and cows with subclinical endometritis	Brodzki et al., 2015 [28]
	Healthy cows and cows with endometritis	Kaya et al., 2016 [29]
	Cows affected by endometritis, before and after treatment	Ahmadi et al., 2018 [30]
	Healthy cows and cows with mastitis	Eckersall et al., 2001 [31]
	Mastitis experimentally induced with Streptococcus uberis	Pedersen et al., 2003 [32]
	Acute and chronic experimentally induced Staphylococcus aureus mastitis	Grönlund et al., 2003 [33]
	Subclinical mastitis in dairy cows	Gerardi et al., 2009 [34]
	Chronic subclinical mastitis in dairy cows Subclinical experimentally induced mastitis with Staphylococcus epidermidis in sheep	Grönlund et al., 2005 [35] Winter et al., 2003 [36]
EQUINE	Before and after insemination	Tuppits et al., 2014 [37]
	Subclinical endometritis	Sikora et al., 2016 [38]
	Before and after artificial insemination in uterine lavage fluid	Wojtysiak et al., 2020 [39]
	Experimentally induced ascending placentitis	Coutinho da Silva et al., 2012 [40]
	Healthy periparturient mares and mares with ascending placentitis	Coutinho da Silva et al., 2013 [41]

**Table 2:** Comparison of concentration of serum amyloid A on serum samples in healthy and disease, considering the literature currently available.

Species	Disease/Condition	Literature/References in Healthy Animals	Literature/References in Disease	Reference
DOG	Pyometra	27.1 ± 13.1 μg/mL	184.2 ± 122.3 μg/mL	Dabrowski et al., 2006 [17]
		<5 μg/mL	61.3 ± 31 (<5–>80) μg/mL	Hagman et al., 2009 [19]
		0.55 ± 0.54 μg/mL	103.56 ± 35.2 μg/mL ^1^ 193.81 ± 20.7 μg/mL ^2^	Dabrowski et al., 2017 [22]
	Mammary tumor	1.69 (1.35–2.05) mg/L	Stage IV 75.87 (38.13–92.76) mg/L	Tecles et al., 2009 [24]
CAT	Pyometra	0 µg/mL	154.8 (0.1–182.4) μg/mL	Yuki et al., 2020 [25]
RUMINANTS	Cattle endometritis	48 ± 20 mg/mL ^3^	85 ± 23 mg/mL ^3^	Chan et al., 2009 [26]
		16.80 ± 1.62 μg/mL	33.97 ± 2.14 to 35.42 ± 0.58 μg/mL	Biswal et al., 2014 [27]
		30 µg/mL ^§,^^4^	50 µg/mL ^§,^^4^	Brodzki et al., 2015 [28]
		14.24 ± 0.52 µg/mL	Mild Endometritis = 20.25 ± 0.65, Endometritis = 28.17 ± 1.22, Severe Endometritis = 34.62 ± 1.28 µg/mL	Kaya et al., 2016 [29]
	Cattle mastitis	5.1 (3.6–11) μg/mL	Mild mastitis = 13.8 (5.4–142) μg/mL Moderate mastitis = 29.9 (5.9–141) μg/mL	Eckersall et al., 2001 [31]
		0.47–4.62 μg/mL ^5^	10-fold increase ^5^	Pedersen et al., 2003 [32]
		2.82 ± 1.8 mg/L	Acute mastitis = 376.9 ± 352 mg/L Chronic mastitis = 11.3 ± 11.0 mg/L	Grönlund et al., 2003 [33]
		50 mg/L ^§^	Subclinical mastitis = 104 mg/L ^§^ Clinical mastitis = 245 mg/L ^§^	Gerardi et al., 2009 [34]
	Sheep mastitis	1.5 (0–29.4) μg/mL	207 μg/mL ^§^	Winter et al., 2003 [36]
EQUINE	Post-insemination	Before: 0.23 (0.05–10.16) mg/L	After: 0.30 (0.02–1.81) mg/L	Tuppits et al., 2014 [37]
		0.001 g/L in uterine lavage fluid ^4^	0.0015 g/L in uterine lavage ^4^	Wojtysiak et al., 2020 [39]

^§^ Average on serum samples; ^1^ open-cervix pyometra; ^2^ closed-cervix pyometra; ^3^ a week after parturition; ^4^ no range was reported within the reference paper; ^5^ evaluated in milk, no data available on serum concentration.

## 2. SAA in Reproductive Disease of Pets

The most common reproductive disorder affecting bitches is pyometra, a severe form of endometritis [42], often caused by *Escherichia coli* (*E. coli*), characterized by pus accumulation into the uterine lumen [43]. The disruption of the cell wall of *E. coli* causes different concatenated events, including the release of lipopolysaccharides (LPS), neutrophils activation, increased synthesis of proinflammatory cytokines [44], systemic inflammation, septic shock, and death [43]. Despite the availability of different medical conservative treatments, ovariohysterectomy remains the treatment of choice. Therefore, a proper monitoring of pre- and post-operative inflammation in bitches with pyometra becomes essential to promptly detect any sub-sequent pyometra complications, such as sepsis and septic shock, systemic inflammatory response syndrome (SIRS), peritonitis, disseminated bacterial infection, and multi-organ dysfunction [45]. The early identification and appropriate treatment of pyometra is crucial to obtain a favorable outcome which can increase the survival rate of this life-threatening condition; consequently, some authors have assessed the changes in serum APPs in bitches with pyometra. In one evaluation [17], Dabrowski et al. found increased serum levels of SAA in pyometra-affected bitches compared to healthy subjects, as a result of the chronic endometrial inflammation. After ovariohysterectomy, SAA levels slowly reduced during the postoperative period in association with the recovery of the bitches. According to their results, the authors concluded that SAA concentrations can be used as markers of inflammation in bitches with pyometra, as demonstrated also by Jiptean et al. [21], who found increased SAA serum concentrations in dogs with pyometra-induced sepsis. The monitoring of SAA levels after ovariohysterectomy can also provide valuable information about inflammatory response during postoperative course as confirmed by another study by Dabrowski et al. [18] who assessed the usefulness of APPs determinations for monitoring the severity of postoperative inflammatory responses in bitches with pyometra undergoing ovariohysterectomy. In that study, serum levels of different APPs, including SAA, remained over the upper limits or even increased in bitches with postoperative complications, compared to animals undergoing a normal post-operative recovery. Considering this fluctuation, authors concluded that SAA may be used as markers for surgical site infections and that determination of serum APPs is a good prognostic indicator which can enable the early detection of bacterial infections of postoperative wounds in bitches that underwent surgery for pyometra.

In another evaluation [22], Dabrowski et al. found that SAA levels reflect the intensity of inflammatory processes in bitches with open- and closed-cervix pyometra regardless of the site of blood collection, suggesting once again the usefulness of evaluating peripheral blood APPs levels as indicators of uterine inflammation, with higher concentrations of APPs in the peripheral blood than in the uterine arterial blood of bitches with closed-cervix pyometra. Similar findings were already noted by Hagman et al. [19], who linked increased SAA concentrations with a strong upregulation of the SAA gene in the uterus, due to inflammatory stimuli.

According to the studies by Dabrowski et al., serum levels of APPs can serve as valid indicators of uterine inflammation in bitches with pyometra providing useful information about the progression of recovery after surgical treatment [19].

Similarly, Christensen et al. found that SAA is useful as a diagnostic marker of systemic inflammation in dogs, including pyometra-related inflammation [20]. Both markers showed comparable diagnostic capacity and achieved and excellent agreement in their performance. Interestingly, Christensen et al. found that SAA was characterized by a wider range of concentrations and a significantly superior overall diagnostic potential for systemic inflammation than other APPs.

APPs has also been evaluated in mammary diseases which represent a frequent health problems of bitches [23]. APPs from serum and milk specimens may be used as accurate inflammatory biomarkers, since their concentrations are significantly higher in bitches suffering from mastitis than in healthy ones [23]. This is particularly important in the case of initial or subclinical mastitis, as standard diagnostic procedures, such as clinical examinations, blood tests, and cytological examinations of mammary gland secretion, may fail to diagnose a condition that, if misdiagnosed or left untreated, can become life-threatening.

SAA fluctuation has also been tested in mammary gland tumors that represent up to 52% of neoplasm in female dogs, they and are diagnosed as malignant in up to 50% of cases [46]. In their work, Tecles et al. [24] found that, in female dogs with mammary gland tumors, APP secretion is dependent upon factors such as the presence of metastasis, the large size of the primary mass, ulceration, or the secondary inflammation of the neoplasm. The preliminary results obtained by Tecles et al. deserves to be further explored to better ascertain a possible application of APPs in the monitoring of therapy and long-term prognosis of mammary tumors in female dogs.

Limited data are available on the evaluation of SAA levels in cats and the possible correlation with diseases [47,48]. Despite this, in a very recent paper, significant increases in SAA serum levels were observed in cats affected by pyometra [25].

## 3. SAA in Ruminants Reproduction

The need for maintaining high-level performance in dairy herds has encouraged researchers to explore suitable indicators of herd health status, which may be represented by APPs [49,50]. In cows, stress-related factors along with damage to the reproductive tract have been shown to be responsible for increasing the concentrations of SAA, suggesting a possible use to monitor the onset of inflammatory and infective disease in this species [26].

Humblet et al. [51] correlated APP concentrations with clinical health status for the diagnosis of disease in the post-parturition period in dairy cattle. The study tested 158 dairy cows from four different herds that underwent clinical and gynecological examination every two weeks over a six-month period; at each time point, serum SAA levels were assessed, and the results were classified as positive or negative based on ad-hoc cutoff points. Although interindividual variability was high, authors found a statistically significant increase in SAA concentrations in the first week after calving, confirming that parturition is associated with a physiologic acute phase response, as previously reported in this species [52,53]. This should be considered when SAA is evaluated as a marker of inflammation within the first week after calving, since it could be challenging to discriminate increases in physiological APPs following normal peripartum from pathological increases related to the onset of inflammatory disease. Authors concluded that, in the postpartum period, SAA was capable of identifying healthy animals; however, its ability to identify animals with ongoing pathologic processes was only fair, and further studies are needed [51].

To better ascertain a possible use of SAA as indicators of uterine infection in dairy cows, Chan et al. [26] tested serum fluctuations over time in cows with metritis. Authors collected blood samples from 18 Holstein dairy cows which displayed acute puerperal metritis at stated intervals from one week prepartum to six months post-partum, using six clinically healthy cows as controls. In addition, authors included ten heifers used to highlight the threshold values of normality for SAA, which was 51.9 mg/mL. SAA concentrations increased significantly in cows after calving, confirming the serum increase in APPs in cows within 3 weeks after parturition, as a laboratory sign of postpartum metritis [54,55,56]. The increase in APPs is primarily due to subclinical endometritis which can seriously reflect its impact on reproductive performance [57]. Reproductive performance in cows with normal and increased APPs concentrations was evaluated by Gilbert et al. [49], taking into account the number of days open and the conception rate. Clinically healthy cows had a better conception rate than those affected by postpartum metritis. Furthermore, among the successfully pregnant cows, the number of days open was significantly higher in cows with Hp values above the threshold limit compared to the remaining cows with a normal range. Interestingly, a significant difference in reproductive performance was not observed between cows with SAA values within or exceeding threshold limit described above.

Biswal et al. [27] evaluated serum levels for APPs at certain time points during the treatment of endometritis to test the possible usefulness of this marker to monitor the treatment progress when using different immunomodulators. Twenty-one cows diagnosed with endometritis underwent three different treatments for this disease, using seven healthy cows as controls, by collecting serum to evaluate SAA levels pre- and post-treatment. SAA decreased post-treatment, irrespective of the type of treatment and according to these results, authors concluded that SAA might serve as reliable biomarkers in the diagnosis and monitoring of endometritis in dairy cows.

Brozki et al. [28] found that even cows affected by subclinical endometritis in the late post-partum period (sixty days after parturition) showed higher serum levels of inflammatory cytokines and APPs in comparison to healthy animals. SAA concentrations increased only in serum while it was not detected in uterine fluid; in fact, the local production of this protein in endometrial cells has only been postulated and not confirmed yet.

A study by Kaya et al. [29] was carried out with the aim of providing a simple and effective tool to assess health herd, by correlating serum SAA levels and serum ceruloplasmin with endometritis which display various degrees of severity in cows. A total of 100 Brown Swiss cows, at 28–32 days postpartum, were divided into two groups—healthy (no endometritis) or endometritis (mild, moderate, and severe) based on ultrasonography, vaginoscopy, and cytology. According to study results, SAA and ceruloplasmin concentrations were significantly higher in cows with endometritis than in healthy controls, and positively correlated with the severity of the endometritis, by confirming the utility of APPs as markers of endometritis in cows.

In 2018, Ahmadi et al. [30] examined the fluctuation of serum SAA in 81 lactating dairy cows affected by postpartum clinical endometritis 30 days after calving. Animals were treated with hyperimmune serum, and the serum concentration of SAA was evaluated before and two weeks after therapy, by showing significant lower levels of this APP following treatment. In addition, the increase in serum levels of SAA was directly correlated to the total milk production per standard lactation (305 days). Ahmadi and collaborators stated that hyperimmune serum administered to treat clinical endometritis could decrease SAA in dairy cows. Interestingly, factors such as milk production and pregnancy status could increase serum levels of SAA.

APPs were found to be useful to assess both acute and subclinical mastitis in dairy cows. SAA milk concentrations were effective indicators of healthy udder quarters; they were within the normal limit in healthy mammary glands and increased in chronic subclinical mastitis [31,32,33,34,35].

Mastitis is a common problem in cattle and sheep herds, representing a significant welfare and financial issue in farming [16]. This is particularly true in the case of subclinical mastitis which is still hard to promptly diagnose [58,59].

In sheep, SAA has been preliminary appraised as a marker of udder health. Winter et al. [36] experimentally induced subclinical mastitis in ewes by inoculation of *Staphylococcus epidermidis* into the udder and then evaluated SAA concentrations in both serum and milk. Although not all the infected ewes showed an increase in serum SAA 24 h after bacteria inoculation, the SAA concentration peaked in milk between 24 and 48 h by returning to levels slightly above the control values within one week. Interestingly, Winter et al. found that basal serum level of SAA in healthy sheep was like those recorded in cattle by Gronlund and collaborators [35]. Starting from these results, Winter et al. further evaluated SAA as a diagnostic marker of subclinical mastitis in ewes, demonstrating that SAA levels in milk, but not in serum, can detect subclinical mastitis in individual ewes [60]. A study by Miglio et al. determined serum levels of SAA in lactating Lacaune sheep [61] of different ages, with the aim of providing a reference interval for SAA in healthy animals.

## 4. SAA in Equids Reproduction

The use of APPs in equine practice has recently raised attention by different research groups with controversial opinions within the scientific community [62,63]. SAA has been one of the most studied APP in several disorders, including those related to reproductive sphere [64,65].

A study [47] monitored the changes in serum SAA during infectious endometritis in mares, correlating these APPs to the local innate immune response. Experimentally induced bacterial endometritis with *E. coli* strains determined a significant increase in SAA, because of an up-regulated endometrial gene expression of SAA, as well as several pro- and anti-inflammatory cytokines [47].

Conversely, other authors found that measurement of SAA was not suggestive of subclinical endometritis in mares, keeping the debate alive [37,38,66,67]. Tuppits et al. [37] investigated changes in serum SAA every 48 h during estrous and 5, 6, or 7 days after artificial insemination (AI) with frozen-thawed semen. Standardbred mares, each with a different reproductive status, were divided in groups accordingly (healthy in first postpartum estrus, healthy barren mares, and mares with endometritis). Post-AI endometritis was determined by bacteriological, cytological, and ultrasonographical examinations. Authors found no statistical difference in SAA levels during the study and after AI, concluding that frozen-thawed insemination did not affect serum APPs. Similar results were obtained by Sikora et al. [38], who found no significant increase in serum SAA of Icelandic mares with subclinical endometritis, thus questioning the usefulness of serum APPs for the diagnosis of uterine inflammation. Conversely, Wojtysiak et al. [39] evaluated the local uterine production of SAA before and after AI, observing a significant increase in SAA concentrations in uterine flushing after AI.

APPs fluctuations have also been investigated in the periparturient period in healthy mares and in mares with placentitis, with the aim to possibly provide a reliable diagnostic tool to identify early onset of placental diseases that represent prevalent causes of abortion, premature delivery, and neonatal death in equine species.

Coutinho da Silva [40] found that serum SAA concentrations significantly increased and remained elevated until abortion due to ascending placentitis experimentally induced by *Streptococcus zooepidemicus*. This study’s results suggest the use of SAA as possible diagnostic aid in spontaneously occurring placentitis during late-term gestation, which represents a hard-to-diagnose condition in clinical practice. In a further study, Coutinho da Silva et al. [41] confirmed these preliminary results. Fifteen healthy pregnant mares were evaluated weekly from 280 days of gestation to foaling and then at 12, 36 and 60 h post-partum. SAA levels remained within normal intervals during pregnancy, apart from three mares that showed higher SAA values the week before foaling and increased significantly at 12 and 36 h following parturition. In the second part of the same study, authors induced placentitis in 14 pregnant mares by intra-cervix inoculation of *Streptococcus zooepidemicus* between 280 and 295 days of gestation, dividing animals into treated (*n* = 9) and control (*n* = 5) groups. SAA concentrations were determined prior to inoculation and then weekly until abortion or parturition. In untreated mares, SAA increased within 96 ± 56 h from bacteria inoculation and remained elevated until abortion, whereas in the treated group, medical treatment maintained SAA levels within the limits in six out of nine mares [41]. Authors concluded that SAA may serve as both a prognostic indicator in cases of ascending placentitis in the mare and a marker to monitor the treatment efficacy. Similarly, Canisso et al. found higher levels of SAA in pregnant mares after the induction of placentitis and until abortion [68].

Changes in APPs have also been evaluated in healthy mares during peripartum by Krakowski et al. [69] who found a certain degree of fluctuations in serum concentrations of SAA. However, the concentration remained within physiological ranges; thus, authors did not consider APPs suitable for indicating potential susceptibility to peripartum disorders in mares.

Although horses are not the only equids among domestic animals, few studies investigated SAA in donkeys and mules [70,71] and only one has assessed SAA levels in healthy jennies and donkey foals in the post-partum period [72]. This study highlighted the effect of parturition on SAA levels, with both jennies and foals giving higher SAA values within 48 h from birth [72].

## 5. Discussion

APPs represent promising diagnostic aids for the early identification of disorders in different fields of veterinary medicine, allowing a continuous monitoring of disease progression and treatment response [4]. However, APPs should not be used as solely indicators of disease and they should be evaluated in adjunction to clinical workup [73]. In animal reproduction, SAA has been proven to be very useful in the detection of challenging diseases, such as subclinical endometritis, with the possibility to provide information regarding the development of the disease [26,74].

The clinical application of SAA evaluation as a routine test has some limitations due to practical reasons. On the one hand, despite the availability of portable devices, the analysis of SAA is time-consuming and still relatively expensive, limiting the wide-scale use of APP evaluation in routine clinical practice [74,75]. Furthermore, the lack of recognized reference ranges for domestic species still limits the use of APPs in clinical practice [74,75]. Despite this, the development and optimization of rapid and economic devices for SAA measurement should be encouraged, considering the broad spectrum of possible applications of acute-phase protein-based diagnostics [75] in veterinary reproduction.

SAA was found to provide diagnostic and prognostic support in the monitoring of the progression of sepsis and post-operative inflammation in subclinical endometritis, endometrial hyperplasia, and pyometra in dogs and cats [12].

Endometritis is one of the first conditions that causes subfertility and infertility in cows, and the possibility to use a reliable indicator could facilitate clinician in both detection and monitoring of such conditions [75,76,77,78].

In small ruminants, SAA can play a crucial role in the prevention of economic losses related to reproductive disorders, especially in rural household environments [79].

In equine medicine, the understanding of serum and endometrial expression of APPs and other cytokines implicated in uterine defense mechanisms could lead to new therapeutic strategies for endometritis, and maybe identifying further diagnostic mediators/markers for the infertility of equids [13,14,80,81,82].

## 6. Conclusions

Due to its rapid increase after the onset of APR, SAA is considered a sensitive and early indicator of inflammation in domestic species. These characteristics make the assessment of SAA an effective diagnostic aid in the field of animal reproduction, since the early detection and prompt treatment of reproductive diseases are essential to preserve fertility. Notwithstanding the usefulness of SAA assessment in animal diseases, further efforts are needed to include this APP in routine clinical pathology screening.

## Data Availability

Not applicable.
